# CircRNA RNA hsa_circ_0008234 Promotes Colon Cancer Progression by Regulating the miR-338-3p/ETS1 Axis and PI3K/AKT/mTOR Signaling

**DOI:** 10.3390/cancers15072068

**Published:** 2023-03-30

**Authors:** Dejun Wu, Yuqin Li, Anjun Xu, Wenqing Tang, Bo Yu

**Affiliations:** 1 Department of General Surgery, Shanghai Pudong Hospital, Fudan University Pudong Medical Center, 2800 Gongwei Road, Shanghai 201399, China; 2 Department of Gastrointestinal Surgery, Shanghai Pudong Hospital, Fudan University Pudong Medical Center, 2800 Gongwei Road, Shanghai 201399, China; 3 Department of Gastroenterology, Shanghai Pudong Hospital, Fudan University Pudong Medical Center, No.2800 Gongwei Road, Pudong New District, Shanghai 201399, China; 4 Department of Gastroenterology and Hepatology, Shanghai Institute of Liver Disease, Zhongshan Hospital, Fudan University, Shanghai 201399, China; 5 Vascular Surgery Department, Shanghai Pudong Hospital, Fudan University Pudong Medical Center, 2800 Gongwei Road, Shanghai 201399, China; 6 Shanghai Key Laboratory of Vascular Lesions Regulation and Remodeling, Shanghai 201399, China

**Keywords:** colon cancer, hsa_circ_0008234, miR-338-3p, ETS1, PI3K

## Abstract

**Simple Summary:**

Circular RNAs (circRNAs) exert an important role in cancer progression. Meanwhile, considering its widespread regulatory effect and the potential of noninvasive testing, understanding the role of circRNA in cancer is particularly meaningful. Our study identified the circRNA hsa_circ_0008234 in colon cancer based on an open-accessed circRNAs expression profile. Moreover, we found that hsa_circ_0008234 could promote proliferation, invasion, and migration abilities of colon cancers, which was partly dependent on the competitive endogenous RNA mechanism (miR-338-3p/ETS1 axis). Meanwhile, we discovered that PI3K/AKT/mTOR signaling is the downstream pathway of the has_circ_0008234/miR-338-3p/ETS1 axis, which improves the effect network of circRNAs in colon cancer.

**Abstract:**

Circular RNAs (circRNAs) have been shown to play a crucial role in cancer occurrence and progression. This present work investigated the link between hsa_circ_0008234 and colon cancer. Data retrieved from GSE172229 was used to compare the circRNA profiles of colon cancer and surrounding non-tumorous tissues. The amount of RNA and protein in the molecules was determined using quantitative real-time PCR (qRT-PCR) and Western blot analysis, respectively. The cell proliferation ability was assessed using CCK8, EdU, colon formation, and nude mice tumorigenesis tests. Cell invasion and migration abilities were evaluated using transwell wound healing and mice lung metastasis model. Hsa_circ_0008234 piqued our interest because bioinformatics and qRT-PCR analyses revealed that it is upregulated in colon cancer tissue. Cell phenotypic studies suggest that hsa_circ_0008234 may significantly increase colon cancer cell aggressiveness. Mice experiments revealed that inhibiting hsa_circ_0008234 significantly reduced tumor growth and metastasis. Moreover, the fluorescence in situ hybridization experiment demonstrated that hsa_circ_0008234 is primarily found in the cytoplasm, implying that it potentially functions via a competitive endogenous RNA pathway. These findings indicated that hsa_circ_0008234 may act as a “molecular sponge” for miR-338-3p, increasing the expression of miR-338-target 3p’s ETS1. In addition, the traditional oncogenic pathway PI3K/AKT/mTOR signaling was found to be the potential downstream pathway of the hsa_circ_0008234/miR-338-3p/ETS1 axis. In conclusion, hsa_circ_0008234 increases colon cancer proliferation, infiltration, and migration via the miR-338-3p/ETS1/PI3K/AKT axis; therefore, it could serve as a target and a focus for colon cancer therapy.

## 1. Introduction

Colon cancer is one of the most prevalent malignant tumors and one of the leading causes of cancer mortality deaths across the globe [1]. Although the mechanism is not fully understood, relevant evidence indicates that the incidence of colon cancer is often affected by multiple factors, such as heavy drinking, poor diet, genetic susceptibility, and so on [2]. Surgery remains the frontline therapy choice for resectable colon cancer, with a 5-year survival rate of nearly 60% in early-stage patients [3]. However, the efficacy of the treatment in advanced stages remains unsatisfactory [4]. In this view, it is critical to examine the unknown mechanisms of colon cancer to provide a theoretical basis for the underlying therapeutic target.

Circular RNA is an endogenous RNA with a special single-stranded closed structure, including exonic circRNAs, exon–intron circRNA, and circularized intron RNA [5]. Exonic circRNAs function primarily in the cytoplasm, whereas intron circRNA function primarily in the nuclei [6]. CircRNA functions in multiple ways, including competitive endogenous RNA (ceRNA), protein interaction, and encoding polypeptides [7]. The abnormal expressions of many circRNA have been observed in cancers, indicating their crucial role in tumor development and genesis [8]. CircRNAs have recently been linked to cancers [9]. Huang et al. discovered that the circRNA hsa_circ_104348 could promote the progression of liver cancer via the miR-187-3p/RTKN2 axis, which relies on the Wnt/β-catenin pathway [10]. Furthermore, Ma et al. discovered that the circRNA hsa_circ_0004872 potentially inhibits cancer progression via the miR-224/Smad4/ADAR1 successive regulatory circuit [11]. Meanwhile, Zheng et al. demonstrated that the circRNA circPPP1R12A encoded a novel protein known as circPPP1R12A-73aa, which could promote colon cancer pathogenesis and metastasis via the Hippo-YAP signaling pathway [12]. Furthermore, Li et al. discovered that circPRRC2A could promote angiogenesis and metastasis in renal cell cancer via the epithelial–mesenchymal transition and activation of TRPM3 [13]. These broad effect patterns of circRNAs make them promising as clinical targets of cancer [14]. While previous research looked into the effect of hsa_circ_0008234 on colon cancer (Table S1) [12,15], the role of the hsa_circ_0008234 function in colon cancer remains unclear.

In this present investigation, we assessed the expression sequence and biological activity of hsa_circ_0008234 in colon cancer. Hsa_circ_0008234 was first identified using the circRNA expression profile of GSE172229, revealing a high expression value in colon cancer tissue and cells. The FISH test revealed predominant levels of hsa_circ_0008234 within the cell cytoplasm, indicating its potential link to the ceRNA pathway. In vitro and in vivo studies revealed that hsa_circ_0008234 might promote the growth, infiltration, and migration of colon cancer. In addition, hsa_circ_0008234 was discovered to potentially increase ETS1 expression via miR-338-3p, which may explain its cancer-promoting activity. The PI3K/AKT/mTOR signaling pathway was revealed as the downstream pathway of the hsa_circ_0008234/miR-338-3p/ETS1 axis.

## 2. Methods and Materials

The circRNA expression profile of colon cancer and adjacent non-tumor was retrieved from the GEO database, GSE172229 (Platform: GPL28148; Agilent-084217 CapitalBio Technology Human CircRNA Array v2 (Beijing, China). Open-accessed transcriptional profiles and clinical information of colon cancer patients were downloaded from The Cancer Genome Atlas (TCGA) database. Differentially expressed circRNAs analysis was performed in the R program (v. 4.0.0) (limma package) with the threshold of |logFC > 1| and *p* value < 0.05. Heatmap was plotted using the heatmap package. Gene set enrichment analysis (GSEA) was performed to explore the biological effect based on Hallmark gene set [16].

### 2.1. Cell Lines Culture and Transfections

The normal colon epithelial cell NCM460 and the four colon cancer cell lines HCT116, SW480, HCT8, and DLD-1, were all laboratory stocks. Cells were cultivated in RPM1640 culture media and replaced every four days. Jima Com (Shanghai, China) provided the short hairpin RNAs (shRNAs) of hsa_circ_0008234 and the control vector, as well as the miR-338-3p, mimics, inhibitor, and ETS1 overexpressed plasmids. Lipofectamine 2000 was used to transfect cells following the standard protocol. The process includes: 1. 5 × 10^5^ cells were inoculated on a 6-well plate, and 2 mL of complete medium was added. Cells converged to 70–90% before transfection; 2. Add 3 μg plasmid into 100 μL serum-free medium and shake it gently; 3. Add 4 μL Lipofectamine 2000 to 100 μL serum-free medium, mix slowly, and leave it at room temperature for 5 min; 4. Mix the diluted Lipofectamine reagent with the plasmid, shake it slowly, and place it at room temperature for 20 min to form the plasmid–Lipofectamine mixture; 5. According to the instructions, add 200 μL plasmid–Lipofectamine mixture to the cell hole containing 800 μL serum-free medium and slowly shake the cell plate; 6. Remove the old medium and add the complete medium after 6 h of culture under conventional conditions (5% CO_2_ and at 37 °C).

### 2.2. Quantitive Real-Time PCR (qPCR) and Sanger Sequencing

Total RNA was extracted by following the instructions provided in the RNA simple Total RNA extraction kit that was manufactured and provided by TIANGEN in Beijing, China. The RNA was then reverse transcribed using a High-Capacity cDNA Reverse Transcription Kit (Themofisher). As a result, total RNA was reverse transcribed into cDNA. The circular nature of the PCR result of hsa_circ_0008234 was determined by Sanger sequencing. RiboBio Co., Ltd. (Guangzhou, China) designed and synthesized the PCR primers. The following primers were used were: Hsa_circ_0008234, Divergent primer, forward: 5′-CCTTCCAAGACCTCCTTAATA-3′, reverse: 5′-TTTCCAGCATGTTGTTGTTG-3′; Convergent primer, forward: 5′-CACCTCAAGTTATCACTCCC-3′, reverse: 5′-GGGCTGAATTGTCAGAAGG-3′; hsa-miR-338-3p, RT primer, 5′-GTCGTATCCAGTGCAGGGTCCGAGGTATTCGCACTGGATACGACCAACAAA-3′, forward, 5′-GGTGGTCCAGCATCAGTGAT-3′, reverse, 5′-CAGTGCAGGGTCCGAGGT-3′; ETS1, forward: 5′-GATAGTTGTGATCGCCTCACC-3′, reverse: 5′-GTCCTCTGAGTCGAAGCTGTC-3′; GAPDH, forward: 5′-ACAACTTTGGTATCGTGGAAGG-3′, reverse: 5′-GCCATCACGCCACAGTTTC-3′.

### 2.3. Western Blot

The total cellular protein was extracted from samples using a total protein extraction kit (Beyotime, Shanghai, China). AKT (1:5000), phospho-AKT (1:3000), mTOR (1:5000), phospho-mTOR (1:5000), PI3K (1:1000), CyclinD1 (1:5000), and β-actin (1:5000) primary antibodies were purchased from Protentech (1:5000). Phospho-PI3K (1:1000) primary antibody was purchased from cell signaling technology (Shanghai, China). Western blot analysis was performed as previously described [17]. Detailed, the process includes: 1. Collect the cell sediment and wash it with PBS 3 times; 2. Prepare cell lysate; 3. The lysate was blown into the cell sediment and lysed on ice for 30 min; 4. Centrifugation for 15 min (4 °C and 12,000 rpm); 5. Determine protein concentration (BCA Kit, Beyotime, Shanghai, China); 6. Protein denaturation; 7. Prepare the SDS-PAGE gel; 8. Protein gel electrophoresis; 9. Protein transfer to PVDF membrane; 10. Protein blocking; 11. Antibody incubation.

### 2.4. Cell Proliferation Assay

The cell proliferation ability was assessed using CCK8, colony formation, and 5-ethyl-2′-deoxyuridine (EdU) assays. A CCK8 assay was performed using a CCK8 instrument (Dojindo, Shanghai, China). Colony formation and EdU test were performed as previously described [17]. For colony formation assay, inoculations of 500 cells per well were performed into 6-plate wells; for CCK8 assay, 2000 cells per well were inoculated into 96 well plates; for EdU assay, a total of 4 × 10^5^ cells were inoculated into each 6-plate well.

### 2.5. Transwell Assay

Cell infiltration and migration potential were assessed using the transwell assay, as previously described [17]. A medium without serum was added to the upper chamber, while a medium with 20% serum was used for the lower chamber. A total of 4 × 10^4^ cells were added into the upper compartment for 12 h. Finally, cells were fixed with 4% formaldehyde and stained with crystal violet.

### 2.6. Wound Healing Assay

The wound-healing assay process was performed as previously described [18]. Inoculated cells were cultured to 85–95% confluency in 6-well plates. The scratch was made using a 200 μL tip. Using a microscope camera, photos of the cell scratches were taken at 0 and 24 h.

### 2.7. Fluorescence In Situ Hybridization (FISH)

The FISH technique was employed to establish where circRNAs are located subcellularly. Briefly, the FISH assay was performed in accordance with the protocol of the FISH Kit (RiboBio). The cells were then repaired, made permeable, and placed in a prehybridization buffer environment for 30 min. The hsa_circ_0008234 FISH probe was incorporated into the cells overnight at room temperature, along with a previously heated hybridization buffer to a higher temperature. DAPI dye was used for nuclear staining.

### 2.8. RNA Immunoprecipitation (RIP)

The RIP assay was performed using an RIP kit provided by Millipore, Burlington, MA, USA. Briefly, 100 μL of RIP wash buffer was prepared for washing, then added and mixed into a 50 μL suspension of magnetic beads. After that, AGO2 and IgG antibodies were applied and incubated for 30 min. The lysates were placed in a beads–antibody complex, and all the tubes were rotated and incubated at 4 °C for 1 night. The immunoprecipitated RNA was investigated using qRT-PCR.

### 2.9. Luciferase Reporter Assay

The hsa_circ_0008234 and ETS1 sequences, in both their wild-type and mutant forms, were cloned into pGL3-control luciferase reporter vectors. The luciferase reporter vectors co-transfection into control and miR-mimics cells employing lipofectamine 2000. The luciferase activities of the luciferase reporter vectors were assessed using a dual-luciferase reporter assay kit (Promega, Beijing, China).

### 2.10. Immunohistochemistry (IHC)

IHC was used to detect Ki67 in the tumor tissues from nude mice. Briefly, tissues of cell xenografts were fixed with 4% formaldehyde for 24 hours and cut into 4 μm paraffin sections. Anti-Ki67 antibody (1:200 dilution) was incubated overnight at 4 °C after sections were treated with 10 mmol/L sodium citrate buffer. Cells with homogeneous Ki67 staining were considered positive.

### 2.11. Animal Models

The xenograft model and tumor metastasis experiment were conducted on five-week-old nude male BALB/c mice. Both control cells (at a concentration of 5 × 10^6^) and treated cells (at a concentration of 5 × 10^6^) were injected into the backs of the naked mice. All mice were executed 25 days following injection, and tumor weights were measured for IHC. The lung metastasis model was used in in vivo metastasis testing. Cells (1 × 10^6^) were infused into mice via the tail veins. Mice were euthanized, and all lungs were retrieved for IHC examination after another 25 days.

### 2.12. Statistical Analysis

The GraphPad Prism 8 and R software (version 4.0.4) were employed for all statistical analyses. All trials were repeated three times, and the results are presented as the mean with the standard deviation. *p* values of less than 0.05 were considered significant.

## 3. Results

### 3.1. CircRNA hsa_circ_0008234 Is Overexpressed in Colon Cancer Tissue and Cell Lines

**Figure 1:**
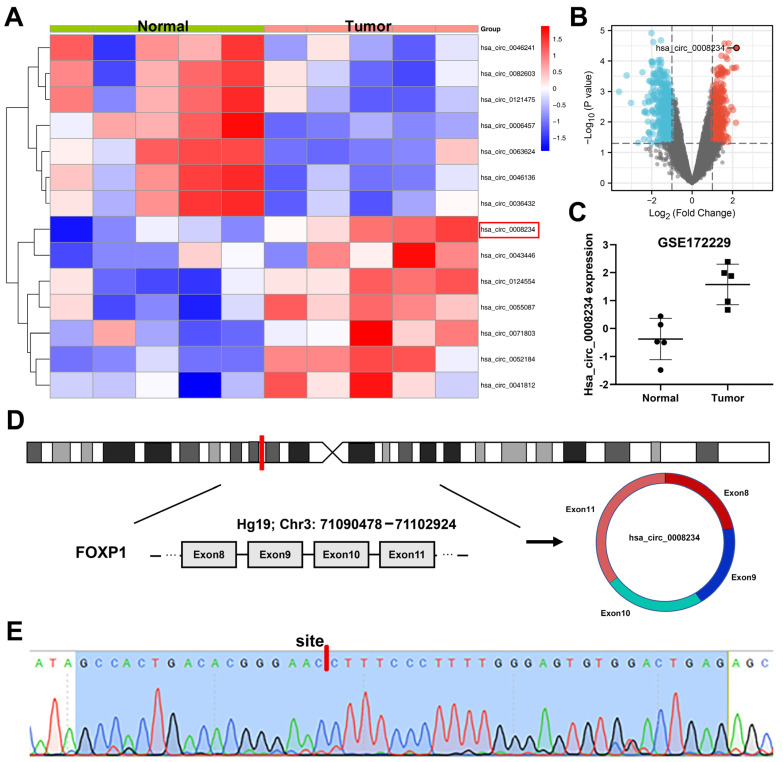
Identification and validation of hsa_circ_0008234 in colon cancer Notes: (**A**): The highest five increased and decreased circRNAs between the normal and colon cancer tissue obtained from the GSE172229. (**B**): Volcano plot of the differentially expressed circRNAs with the threshold of |logFC| > 1 and *p* < 0.05. (**C**): Hsa_circ_0008234 was upregulated in the colon cancer tissue based on the GSE172229. (**D**): The genomic position of hsa_circ_0008234. (**E**): The RT-PCR results of has_circ_0008234 were sequenced using the Sanger sequencing method. The location of the splice is highlighted by the black arrow of hsa_circ_0008234.

**Figure 2:**
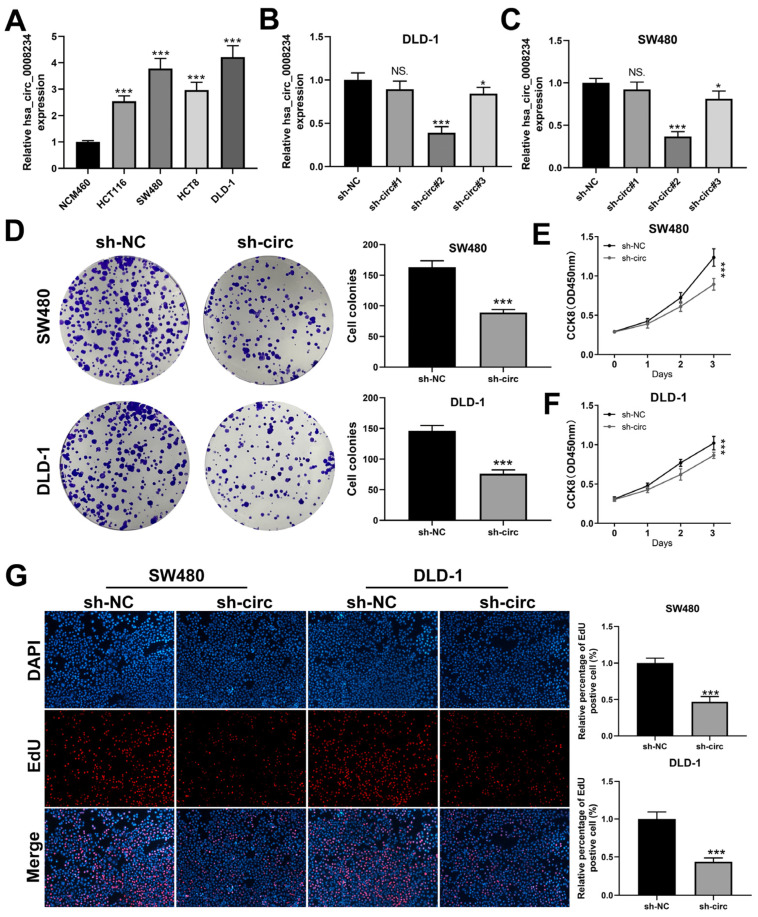
Hsa_circ_0008234 promotes colon cancer proliferation. Notes: (**A**) Hsa_circ_0008234 was increased in colon cancer cells, *** = *p* < 0.001; (**B**,**C**) qRT-PCR assay was conducted to assess the knockdown efficiency of hsa_circ_0008234, NS. = *p* > 0.05, * = *p* < 0.05, *** = *p* < 0.001; (**D**) Colony formation assay between hsa_circ_0008234 knockdown and control cells, *** *p* < 0.001; (**E**,**F**) CCK8 assay between hsa_circ_0008234 knockdown and control cells, *** = *p* < 0.001; and (**G**) EdU assay between hsa_circ_0008234 knockdown and control cells, *** = *p* < 0.001.

Based on the circRNA throughput data of GSE172229, differentially expressed circRNAs were evaluated between five colon cancer and adjacent normal tissues. Figure 1A depicts the top five increased and decreased circRNAs in tumor tissue. Of note, hsa_circ_0008234 was the most significantly upregulated circRNA (Figure 1B). Meanwhile, hsa_circ_0008234 was highly expressed in colon cancer tissue (Figure 1C). Hsa_circ_0008234 includes 587 nucleotides and is derived from the exons 8, 9, 10, and 11 of FOXP1 in chr3 (71090478-71102924), also known as circFOXP1 (Figure 1D). As such, we performed Sanger sequencing of its PCR product to confirm that hsa_circ_0008234 had a covalently closed circular structure (Figure 1E). Moreover, hsa_circ_0008234 was found to have a higher expression value in colon cancer cell lines (Figure 2A).

### 3.2. CircRNA hsa_circ_0008234 Promote the Proliferation, Invasion, and Migration of Colon Cancer Cells

**Figure 3:**
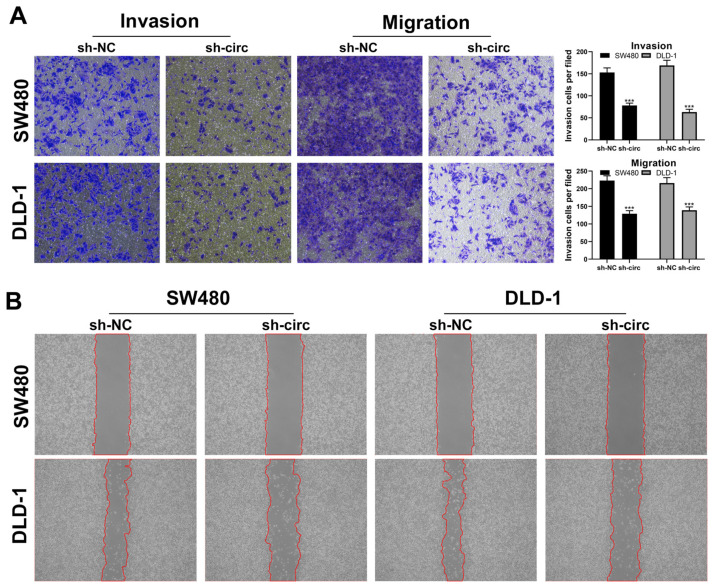
Hsa_circ_0008234 facilitates colon cancer invasion and migration. Notes: (**A**) Transwell assay between hsa_circ_0008234 knockdown and control cells, *** = *p* < 0.001; and (**B**) Wound healing assay between hsa_circ_0008234 knockdown and control cells.

To examine the biological function of hsa_circ_0008234 in colon cancer, we knocked it down and performed qRT-PCR to validate the effectiveness of this knockdown in colon cancer cells. The results indicated that sh-circ#2 was the most efficient, so it was chosen for future experiments (Figure 2B,C and Figure S1A). The SW480, DLD-1, and HCT8 cell lines were used for further experiments due to their higher hsa_circ_0008234 expression. Colony generation and CCK8 experiment demonstrated that hsa_circ_0008234 knockdown significantly reduced colon cell growth (Figure 2D–F and Figure S1B,C). EdU assay results suggested that inhibiting hsa_circ_0008234 significantly decreased the proportion of EdU-positive cells (Figure 2G). Transwell and wound healing tests revealed that hsa_circ_0008234 knockdown significantly suppressed colon cancer cells aggression (Figure 3A,B and Figure S1D–F).

### 3.3. CircRNA hsa_circ_0008234 Functions as a miR-338-3p Sponge in Colon Cancer Cells

**Figure 4:**
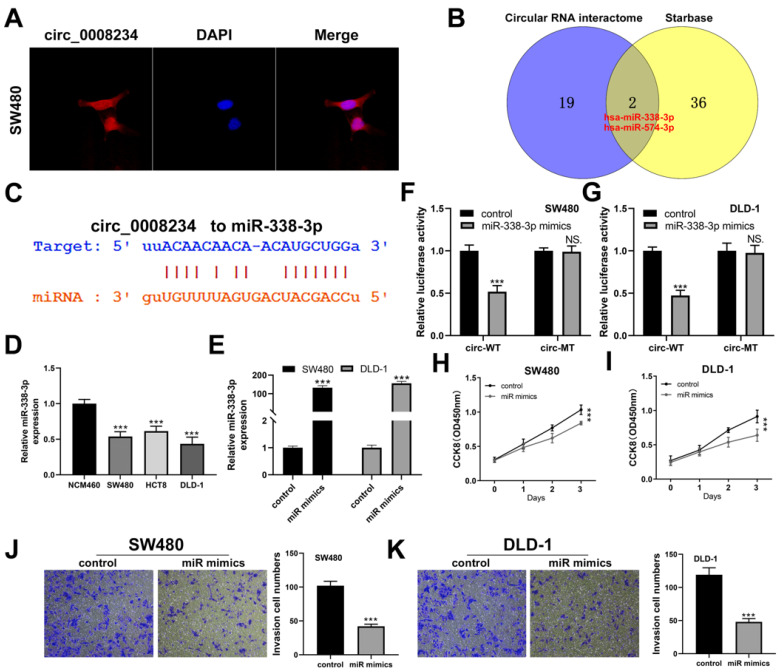
Hsa_circ_0008234 acts as a “molecular sponge” for miR-338-3p. Notes: (**A**) FISH assay of hsa_circ_0008234 in SW480 cell line; (**B**) Circular RNA interactome and Starbase identified two miR-338-3p and miR-574-3p that might be bound with hsa_circ_0008234; (**C**) The docking site of miR-338-3p and hsa_circ_0008234; (**D**) MiR-338-3p was downregulated in colon cancer cells, *** = *p* < 0.001; (**E**) qRT-PCR was utilized to evaluate the overexpression efficiency of miR-338-3p, *** = *p* < 0.001; (**F**,**G**) Luciferase enzyme reporter assay was conducted in the cells harboring wild-type, as well as mut-type of hsa_circ_0008234, NS. = *p* > 0.05, *** = *p* < 0.001; (**H**,**I**) CCK8 testing was done between the miR-338-3p overexpressed and control cells, *** = *p* < 0.001; and (**J**,**K**) Transwell testing was done between the miR-338-3p overexpressed and control cells, *** = *p* < 0.001.

CircRNA subcellular localization can reveal its function mechanism. As such, we employed the FISH test to determine the subcellular localization of hsa_circ_0008234. Hsa_circ_0008234 was found mostly in the cytoplasm, indicating that it may function via a ceRNA mechanism (Figure 4A). Additionally, miRNAs with potential attachment sites for hsa_circ_000824 were discovered using the Circular RNA interactome and Starbase databases (Figure 4B). The miR-338-3p was selected for future research based on previous findings for colon cancer [19]. Figure 4C depicts the anticipated attaching positions of hsa_circ_0008234 and hsa-miR-338-3p. Cell line studies revealed that hsa_miR-338-3p was decreased in colon cancer cells (Figure 4D). Furthermore, we used miR-338-3p mimics, and the qRT-PCR results showed a satisfactory overexpression efficiency (Figure 4E). Transfection of cells with miR-338-3p mimics significantly lowered wild-type hsa_circ_0008234 reporter gene activity while having no effect on hsa_circ_0008234 mutated-type reporter gene activity (Figure 4F,G). CCK8 test revealed that miR-338-3p significantly inhibits colon cancer cell proliferation (Figure 4H,I). Additionally, the transwell test result revealed that miR-338-3p significantly suppressed colon cancer cell aggressiveness (Figure 4 J,K).

### 3.4. ETS1 Is the Targeted Gene of miR-338-3p

**Figure 5:**
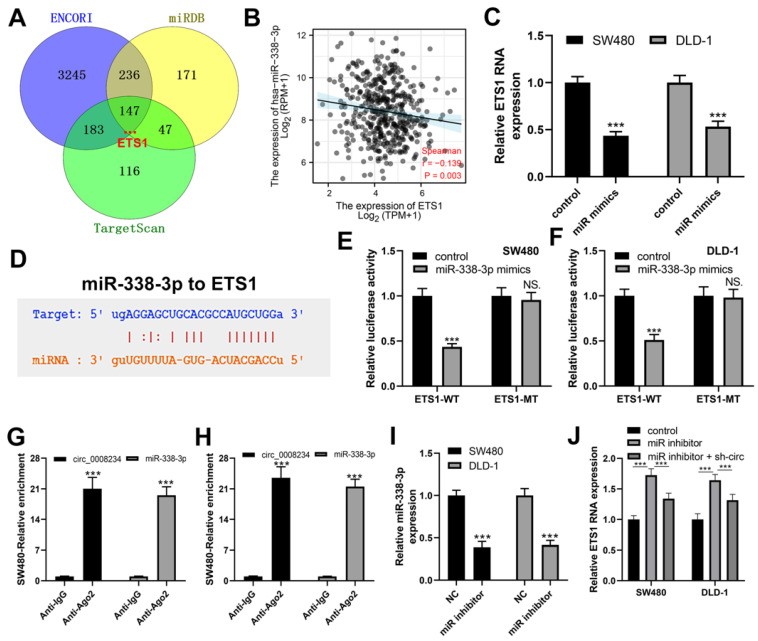
ETS1 is a target gene of miR-338-3p. Notes: (**A**) ENCORI, miRDB, and TargetScan identified ETS1 as the miR-338-3p targeted gene; (**B**) ETS1 exhibited a positive correlation with miR-338-3p according to the TCGA data; (**C**) The ETS1 mRNA expression in miR-338-3p overexpressed and control cells, *** = *p* < 0.001; (**D**) The docking site of miR-338-3p and ETS1; (**E**,**F**) Luciferase enzyme reporter test was conducted in the cells harboring wild-type, as well as mut-type of ETS1, NS. = *p* > 0.05, *** = *p* < 0.001; (**G**,**H**) RIP result demonstrated that hsa_circ_0008234 and miR-338-3p could be significantly supplemented with the AGO2 antibody compared with the control IgG antibody, *** = *p* < 0.001; (**I**) qRT-PCR was conducted to assess the knockdown efficiency of miR-338-3p, *** = *p* < 0.001; and (**J**) The ETS1 mRNA level in differently treated cells, *** *p* < 0.001.

Three miRNA-mRNA databases ENCORI, miRDB and TargetScan were used to identify the target gene for miR-338-3p. miR-338-3p served as an oncogene in colon cancer (Figure 5A) [20]. ETS1 was discovered as the targeted gene for miRNA. Moreover, miR-338-3p was found to be negatively correlated with ETS1 (Figure 5B, r = −0.139, *p* = 0.003). We discovered that cells containing miR-338-3p mimics expressed lower ETS1 RNA values at the cellular level (Figure 5C). The anticipated ETS1 and hsa-miR-338-3p attaching locations were shown in Figure 5D. The luciferase reporter experiment revealed that cells transfected with miR-338-3p mimics had significantly lower ETS1 reporter gene activity in wild-type cells than in ETS1 mutant cells (Figure 5E,F). The association with the AGO2 protein was perceived as a significant indicator of ceRNA function. The RIP results demonstrated that the AGO2 antibody significantly increased hsa_circ_0008234 and miR-338-3p in comparison to the control IgG antibody (Figure 5G,H). The inhibitor efficiency was validated using qRT-PCR (Figure 5I). Moreover, cells treated with miR-338-3p inhibitor had higher ETS1 RNA expression than control cells, but hsa_circ_0008234 knockdown could reverse this effect (Figure 5J).

### 3.5. CircRNA hsa_circ_0008234 Regulates the PI3K/AKT/mTOR Signaling Pathway via the miR-338-3p/ETS1 Axis to Promote the Proliferation, Invasion, and Migration of Colon Cancer

**Figure 6:**
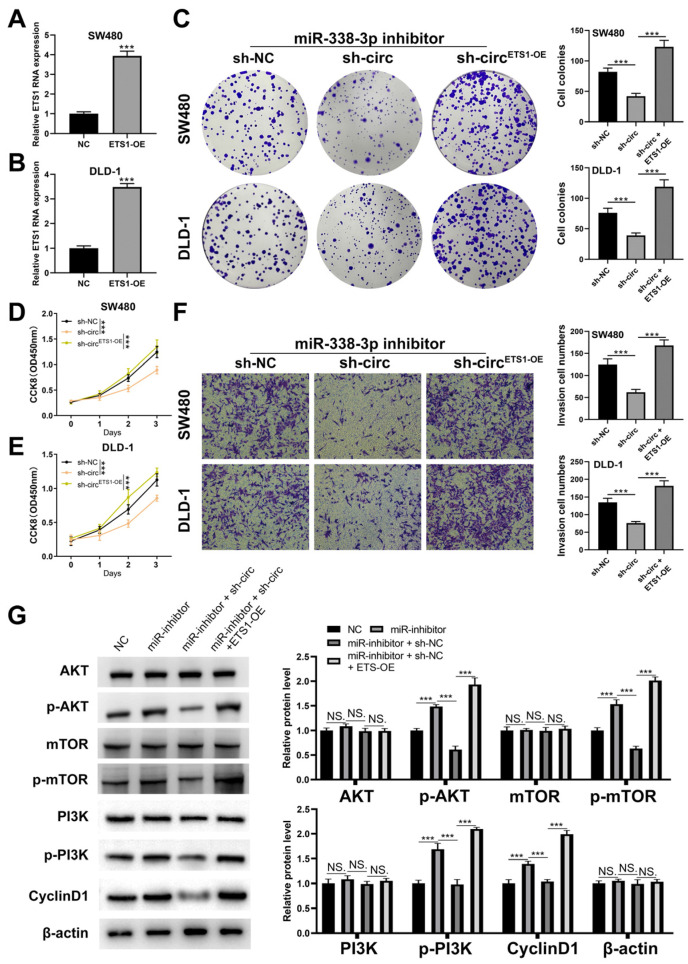
Hsa_circ_0008234 promotes colon cancer progression through miR-338-3p/ETS1/PI3K/AKT axis. Notes: (**A**,**B**) qRT-PCR was utilized to evaluate the overexpression ETS1 efficiency, *** = *p* < 0.001; (**C**) Colony formation assay in cells with miR-338-3p inhibitor, miR-338-3p inhibitor + sh_circ_0008234, and miR-338-3p inhibitor + sh_circ_0008234 + ETS1-OE, *** = *p* < 0.001; (**D**,**E**) CCK8 assay in cells with miR-338-3p inhibitor, miR-338-3p inhibitor + sh_circ_0008234, and miR-338-3p inhibitor + sh_circ_0008234 + ETS1-OE, *** = *p* < 0.001; (**F**) Transwell assay in cells with miR-338-3p inhibitor, miR-338-3p inhibitor + sh_circ_0008234, and miR-338-3p inhibitor + sh_circ_0008234 + ETS1-OE, *** = *p* < 0.001; and (**G**) Western blot assay assessing the key molecules of PI3K/AKT/mTOR pathway was performed in cells with miR-338-3p inhibitor, miR-338-3p inhibitor + sh_circ_0008234, and miR-338-3p inhibitor + sh_circ_0008234 + ETS1-OE, NS. = *p* > 0.05m *** = *p* < 0.001.

We successfully overexpressed ETS1 in treated colon cancer cells (Figure 6A,B). Colony formation revealed that sh-hsa_circ_0008234 inhibited cell proliferation in miR-338-3p inhibitor cells, but ETS1 overexpression alleviated this effect and was rescued by ETS1 overexpression (Figure 6C). The CCK8 assay revealed the same trend (Figure 6D,E). Transwell assays revealed that sh-hsa_circ_0008234 could promote the cell invasion ability of miR-338-3p inhibitor cells. Nevertheless, ETS1 overexpression could rescue this effect (Figure 6F). Evidence from previous research indicates that ETS1 may influence the activity of the PI3K/AKT/mTOR signaling pathway. Additionally, based on TCGA data, GSEA analysis showed that in the patients with high ETS1 expression, the activity of PI3K/AKT/mTOR signaling increased (Figure S2). In this view, we shifted our focus to determining whether the PI3K/AKT/mTOR signaling pathway was related to the downstream pathway of the hsa_circ_0008234/miR-338-3p/ETS1 axis (Figure 6G and Figure S1G). The results revealed that the miR-338-3p inhibitor could increase p-AKT and p-mTOR protein levels, whereas that sh-hsa circ 0008234 could reverse this effect. In addition, ETS1 overexpression may increase the protein values of p-AKT and p-mTOR but not AKT, mTOR, or β-actin levels. Along with the PI3K/AKT/mTOR signaling pathway, the hsa_circ_0008234/miR-338-3p/ETS1 axis may influence the activity of colon cancer malignancy.

### 3.6. CircRNA hsa_circ_0008234 Promotes Colon Cancer Proliferation and Metastasis in Vivo

**Figure 7:**
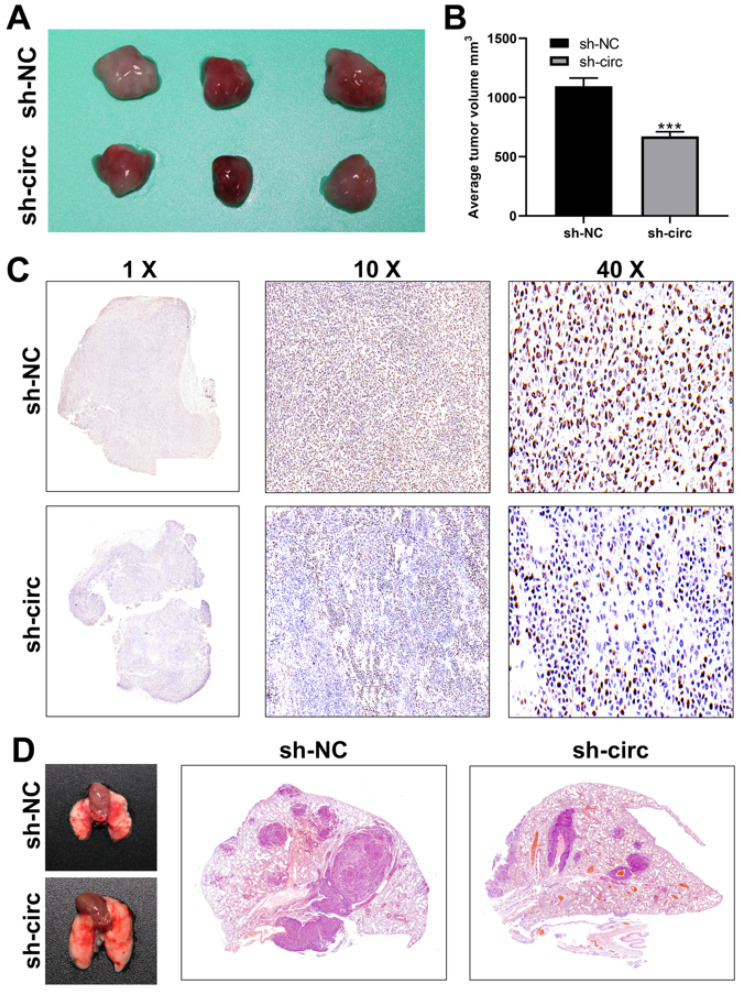
Hsa_circ_0008234 promotes colon cancer growth and metastasis in vivo. Notes: (**A**,**B**) In vivo investigations illustrated that the knockdown of hsa_circ_0008234 significantly dampened tumor proliferation in mice, *** = *p* < 0.001; (**C**) Ki67 staining depicted that the xenograft tumors of sh-hsa_circ_0008234 had a lower proportion of Ki67 positive cells relative to the sh-NC cells; and (**D**) The inhibition of hsa_circ_0008234 could reduce the number of metastatic lung foci.

**Figure 8:**
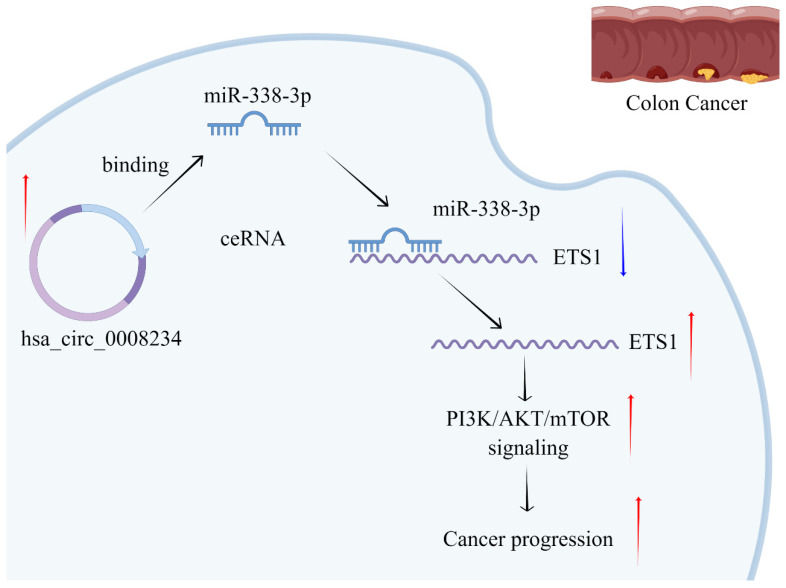
The putative diagram of our study.

The biological effect of hsa_circ0008234 was investigated in vivo. The nude mice tumorigenesis test revealed that hsa_circ_0008234 knockdown could significantly inhibit colon cancer cell growth in vivo (Figure 7A,B). Ki67 staining showed that the sh-hsa_circ_0008234 xenograft tumors had a lower proportion of Ki67-positive cells than sh-NC cells (Figure 7C). The lung metastasis model showed that inhibiting hsa_circ_0008234 could reduce the number of metastatic lung foci (Figure 7D). The concept of our study was shown in Figure 8.

## 4. Discussion

Colon cancer remains a major global public health concern, claiming approximately 600,000 death lives each year [21]. CircRNA has recently received attention due to its broad impact on disease. Meanwhile, the highly stable characteristics brought by its covalent closed-loop structure make circRNA an excellent biomarker for diagnosis and therapy [22]. Consequently, it is urgent and meaningful to explore novel circRNAs that have the potential to be new clinical biomarkers in colon cancer.

In this present investigation, we identified has_circ_0008234 as a potential circRNA for subsequent analyses. CircRNA hsa_circ_0008234 is derived by backward splicing of FOXP1 (forkhead box P1) exons 8, 9, 10, and 11. Elevated hsa_circ_0008234 levels were found in colon cancer cells. In vitro and in vivo tests revealed that hsa_circ_0008234 promoted colon cancer aggressiveness. In particular, hsa_circ_0008234 acts as a miRNA-sponge for miR-338-3p to increase ETS1 RNA expression, which potentially influences AKT/mTOR pathway stimulation. Previous research investigated the biological effects of hsa_circ_0008234 on cancer. For instance, Cai et al. discovered that hsa_circ_0008234 promoted the growth of cutaneous squamous cell carcinoma via the miR-127-5p/ADCY7 axis [23]. Luo et al. demonstrated that has_circ_0008234 could be a unique predictive serum biomarker for non-small cell lung cancer [24]. To the best of our knowledge, this is a pioneer study of the effect of hsa_circ_0008234 in colon cancer.

Recent research suggests that the ceRNA is the primary function mechanism for circRNAs found in the cytosol [25]. According to the FISH results, we discovered that hsa_circ_0008234 was largely localized in the cytosol, indicating that it potentially functions via a ceRNA mechanism. In this view, we searched three databases, ENCORI, miRDB, and TargetScan, to find the miRNAs of interest. Finally, we were intrigued by the miR-338-3p. MiR-338-3p has been shown to influence cancer progression in a variety of cancers. Luan et al. discovered that lncRNA XLOC 006390 could potentially promote tumorigenesis and metastasis of cervical cancer by acting as a ceRNA versus miR-338-3p [26]. In addition, Zou et al. revealed that miR-338-3p could hamper colon cancer by inhibiting MACC1 expression [19]. In our investigation, we found that miR-338-3p was potentially correlated with the cancer-boosting effect of hsa_circ_0008234, which refined the regulatory network of miR-338-3p.

ETS1 is a member of the ETS transcription factors that can recognize the core consensus DNA sequence of GGAA/T in specific genes (ETS-binding domain) [27]. The ETS transcription factor family, which includes ETS1, plays a role in stem cell generation, cell senescence, apoptosis, and tumorigenesis [27]. These ETS family members can either activate or inhibit the transcription process in several different genes. Chen et al. found that WTAP could accelerate the development of hepatocellular carcinoma by suppressing ETS1 via an m6A-HuR-dependent mechanism [28]. Furthermore, Rodgers et al. discovered that ETS1 may activate the transformed β signaling growth factor and promote epithelial-mesenchymal transition in prostate cancer [29]. Gu et al. found that miR-532-3p reduced the development of colorectal cancer by disrupting the ETS1/TGM2-axis-mediated Wnt/β-catenin signaling [30]. We performed several tests in the course of our research to investigate the existing has_circ_0008234 and the miR-338-3p/ETS1 axes relationship. The results of rescue studies suggested that has_circ_0008234 influences the malignancy of colon cancer by regulating ETS1 via miR-338-3p.

Research evidence suggests that ETS1 may accelerate the development of cancer by influencing the function of the PI3K/AKT/mTOR signaling pathway. Xu et al. discovered that miR-129 could inhibit prostate cancer and metastasis by blocking ETS1 and modulating the PI3K/AKT/mTOR pathway [31]. In addition, Tong et al. found that miR-365 inhibits lung cancer development by blocking ETS1 and inactivating the AKT/mTOR pathway [32]. In this present investigation, PI3K/AKT/mTOR signaling was found to be the downstream path of the hsa_circ_0008234/miR-338-3p/ETS1 axis, broadening the regulatory network of PI3K/AKT signaling in cancers. Our results also indicated that hsa_circ_0008234 could regulate the PI3K/AKT/mTOR signaling pathway. As a widespread regulator, circRNA seems reasonable to influence cancer progression by regulating the classic PI3K/AKT/mTOR signaling. Wang et al. discovered that circEPSTI1 could affect oral squamous cell carcinoma progression via PI3K/AKT/mTOR signaling, which is dependent on the mir-942-5p/LTBP2 axis [33]. Moreover, Ling et al. noticed that Circ-PRKDC could promote autophagy and apoptosis in T-cell acute lymphoblastic leukemia via the PI3K/AKT/mTOR signaling [34].

Some limitations should be noticed. Firstly, we only proved the in vivo cancer-promoting effect of hsa_circ_0008234. However, the in vivo experiments of hsa_circ_0003596/miR-502-5p/IGF1R/PI3K/AKT axis have not been performed. Comprehensive in vivo experiments can enhance the stability of the conclusion. Secondly, the evaluation of the tissue level of hsa_circ_0008234 is limited in our study. In the future, a larger sample size of colon cancer is needed to support our conclusions. Thirdly, since circRNAs have a broad regulatory effect, including protein–protein interaction and protein encoding, other underlying mechanisms have not been identified.

## 5. Conclusions

In conclusion, this work revealed the tumor-promoting effect and potential molecular mechanisms of the circRNA hsa_circ_0008234 in colon cancer. Significantly elevated circRNA hsa_circ_0008234 levels in colon cancer indicated that it could play a pivotal part in the diagnosis and prognosis prediction. Moreover, results from in vitro and in vivo investigations demonstrate that hsa_circ_0008234 may promote colon cancer aggressiveness. These findings suggest that hsa_circ_0008234 could be a therapeutic target for colon cancer.

## Data Availability

The circRNA profile data can be obtained from https://www.ncbi.nlm.nih.gov/geo/query/acc.cgi?acc=GSE172229 (accessed on 23 March 2022). The datasets generated and/or analyzed during this current study are not publicly available due to the need for further research but are available from the corresponding author upon reasonable request.

## Supplementary Materials

The following supporting information can be downloaded at: https://www.mdpi.com/article/10.3390/cancers15072068/s1, Figure S1: Role of circ_hsa_0008234 in HCT-8 cell line; Figure S2: GSEA analysis of ETS1 based on TCGA database; Table S1 [12,15,35,36,37,38,39,40,41,42,43,44]: Some circRNAs studies in colon cancer.

## Abbreviations

circRNAs	Circular RNAs
qRT-PCR	Quantitative real-time PCR
shRNAs	Short hairpin RNAs
EdU	5-Ethynyl-2′-deoxyuridine
FISH	Fluorescence in situ hybridization
RIP	RNA immunoprecipitation
IHC	Immunohistochemistry
GEO	Gene Expression Omnibus
