# Supplementary material for: CircRNA RNA hsa_circ_0008234 Promotes Colon Cancer Progression by Regulating the miR-338-3p/ETS1 Axis and PI3K/AKT/mTOR Signaling
Source: Cancers (Basel). 2023 Mar 30;15(7):2068. doi: 10.3390/cancers15072068 (PMC10093195; doi:10.3390/cancers15072068)
Supplement: Supplementary file 1 [file cancers-15-02068-s001.zip › cancers-2158475-supplementary.pdf]

cancers MDPI

Supplementary Materials

CircRNA RNA hsa_circ_0008234 Promotes Colon Cancer Progression by Regulating the miR-338-3p/ETS1 Axis and PI3K/AKT/mTOR Signaling

Table S1. Some CircRNAs in Colon Cancer

circRNA ID	Dysregulation	Mechanism	Reference (PMID)
circPPP1R12A	Up	protein encoded; Hippo-YAP signaling	30925892 [1]
circFNDC3B	Down	protein encoded; EMT	32241279 [2]
circPLOD2	Up	ceRNA; Warburg effect	36071678 [3]
circMETTL3	Down	ceRNA	35710754 [4]
circPPFIA1s	Down	ceRNA; HuR/RAB36 pathway	36224588 [5]
circRNA_104916	Down	EMT	30844715 [6]
hsa_circRNA_000166	Up	ceRNA	32606768 [7]
circRNA_100859	Up	ceRNA	32644049 [8]
circ_0085315	Up	ceRNA	35230926 [9]
circCSPP1	Up	ceRNA	35101080 [10]
hsa_circ_0020095	Up	ceRNA	33520987 [11]
circ_0038718	Up	ceRNA; Wnt/β-catenin signaling	34706645 [12]

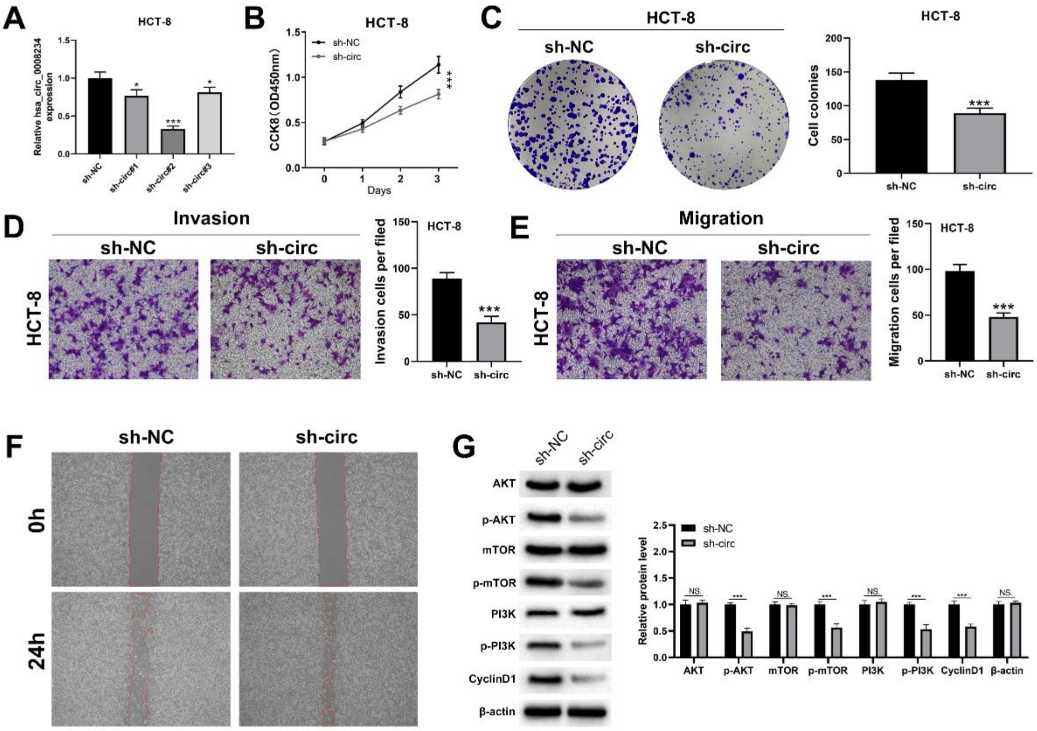

Figure S1. Role of circ_hsa_0008234 in HCT8 cell line.

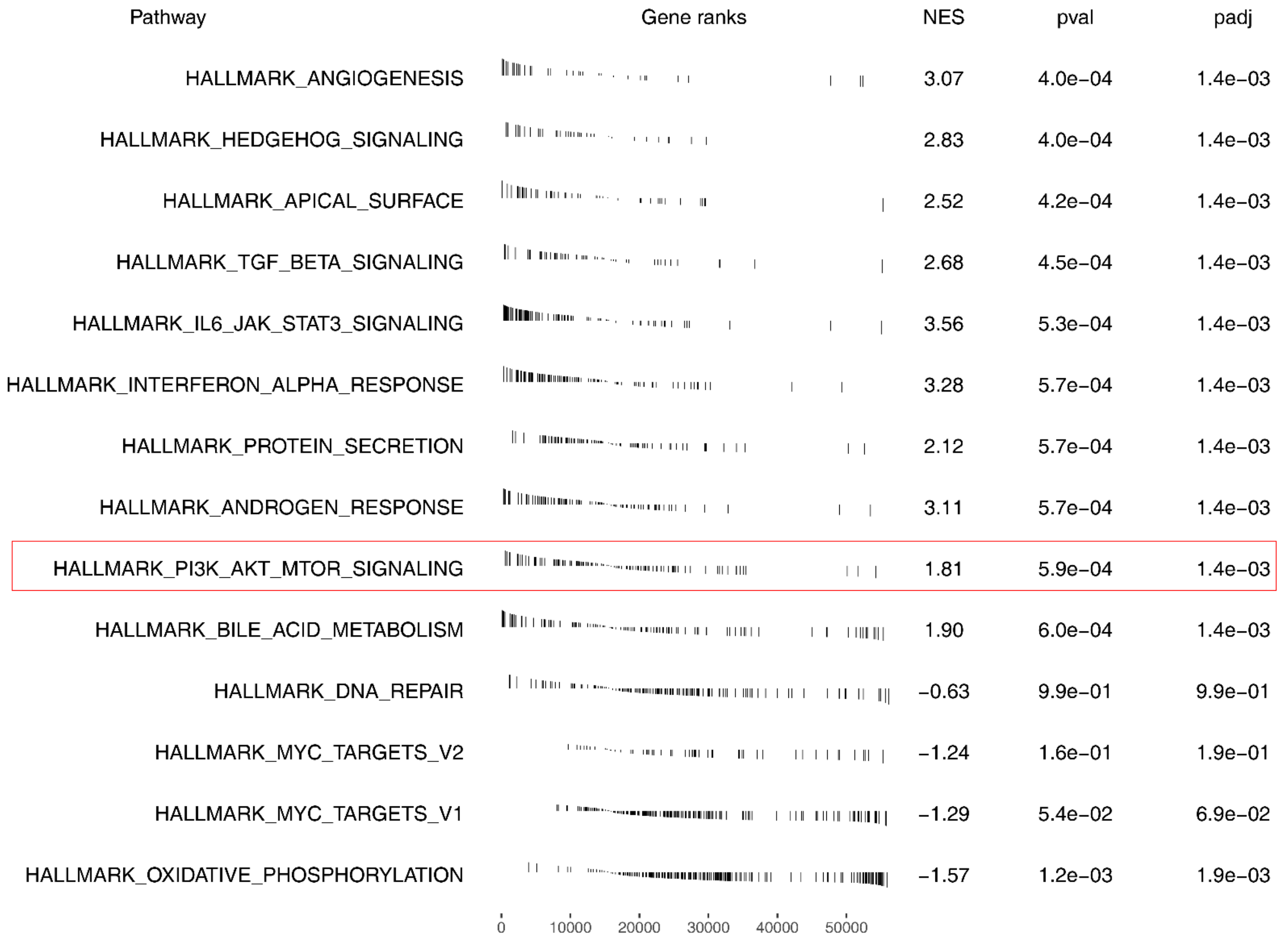

Figure S2. GSEA analysis of ETS1 based on TCGA database.

References

1. Zheng, X.; Chen, L.; Zhou, Y.; Wang, Q.; Zheng, Z.; Xu, B.; Wu, C.; Zhou, Q.; Hu, W.; Wu, C.; et al. A novel protein encoded by a circular RNA circPPP1R12A promotes tumor pathogenesis and metastasis of colon cancer via Hippo-YAP signaling. *Mol. Cancer* **2019**, *18*, 47.
2. Pan, Z.; Cai, J.; Lin, J.; Zhou, H.; Peng, J.; Liang, J.; Xia, L.; Yin, Q.; Zou, B.; Zheng, J.; et al. A novel protein encoded by circFNDC3B inhibits tumor progression and EMT through regulating Snail in colon cancer. *Mol. Cancer* **2020**, *19*, 71.
3. Li, Y.; H. Ma. "Circrna Plod2 Promotes Tumorigenesis and Warburg Effect in Colon Cancer by the Mir-513a-5p/Six1/Ldha Axis." *Cell Cycle*. **2022**, 2484-98.
4. Zhang, F., T. Su, and M. Xiao. "Runx3-Regulated Circrna Mettl3 Inhibits Colorectal Cancer Proliferation and Metastasis Via Mir-107/Per3 Axis." *Cell Death Dis* 13, no. 6 (2022): 550.
5. Ji, H., T. W. Kim, W. J. Lee, S. D. Jeong, Y. B. Cho, and H. H. Kim. "Two Circppfia1s Negatively Regulate Liver Metastasis of Colon Cancer Via Mir-155-5p/Cdx1 and Hur/Rab36." *Mol Cancer* 21, no. 1 (2022): 197.
6. Min, L., H. Wang, and Y. Zeng. "Circrna_104916 Regulates Migration, Apoptosis and Epithelial-Mesenchymal Transition in Colon Cancer Cells." *Front Biosci (Landmark Ed)* 24, no. 5 (2019): 819-32.
7. Zhao, G., and G. J. Dai. "Hsa_Circrna_000166 Promotes Cell Proliferation, Migration and Invasion by Regulating Mir-330-5p/Elk1 in Colon Cancer." *Onco Targets Ther* 13 (2020): 5529-39.
8. Zhou, P., W. Xie, H. L. Huang, R. Q. Huang, C. Tian, H. B. Zhu, Y. H. Dai, and Z. Y. Li. "Circrna_100859 Functions as an Oncogene in Colon Cancer by Sponging the Mir-217-Hif-1α Pathway." *Aging (Albany NY)* 12, no. 13 (2020): 13338-53.
9. Luo, Y., and Q. Yao. "Circ_0085315 Promotes Cell Proliferation, Invasion, and Migration in Colon Cancer through Mir-1200/Map3k1 Signaling Pathway." *Cell Cycle* 21, no. 11 (2022): 1194-211.
10. Wang, J., L. Zhou, B. Chen, Z. Yu, J. Zhang, Z. Zhang, C. Hu, Y. Bai, X. Ruan, S. Wang, J. Ouyang, A. Wu, and X. Zhao. "Circular Rna Circcspp1 Promotes the Occurrence and Development of Colon Cancer by Sponging Mir-431 and Regulating Rock1 and Zeb1." *J Transl Med* 20, no. 1 (2022): 58.

11. Sun, Y., Z. Cao, J. Shan, Y. Gao, X. Liu, D. Ma, and Z. Li. "Hsa_Circ_0020095 Promotes Oncogenesis and Cisplatin Resistance in Colon Cancer by Sponging Mir-487a-3p and Modulating Sox9." *Front Cell Dev Biol* 8 (2020): 604869.
12. Gu, H., Z. Xu, J. Zhang, Y. Wei, L. Cheng, and J. Wang. "Circ_0038718 Promotes Colon Cancer Cell Malignant Progression Via the Mir-195-5p/Axin2 Signaling Axis and Also Effect Wnt/B-Catenin Signal Pathway." *BMC Genomics* 22, no. 1 (2021): 768.

Disclaimer/Publisher's Note: The statements, opinions and data contained in all publications are solely those of the individual author(s) and contributor(s) and not of MDPI and/or the editor(s). MDPI and/or the editor(s) disclaim responsibility for any injury to people or property resulting from any ideas, methods, instructions or products referred to in the content.
